# Supplementary figures and images for: New alleles of D-2-hydroxyglutarate dehydrogenase enable studies of oncometabolite function in Drosophila melanogaster
Source: bioRxiv. 2025 May 22:2025.03.27.645621. Originally published 2025 Apr 1. Preprint. [Version 2] doi: 10.1101/2025.03.27.645621 (PMC11996423; doi:10.1101/2025.03.27.645621)

## Control vs *D2hgdh*<sup>5-5</sup>

### KEGG - Enrichment Overview (Top 25)

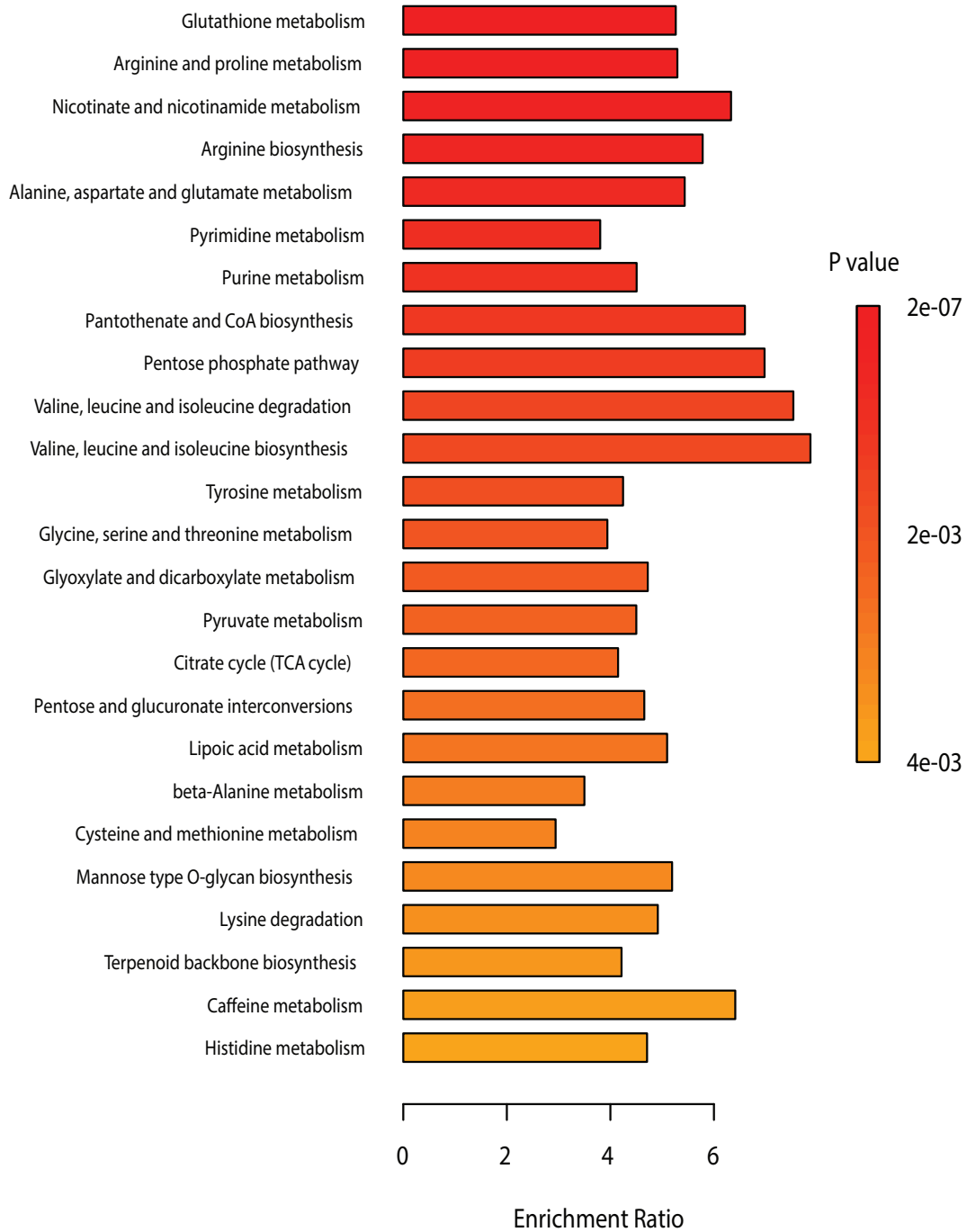

**Figure S2**

Supplement: Supplement 2 — Figure S2. Enrichment analysis of D2hgdh12–6 mutants in comparison to controls. MetaboAnalyst was used to perform KEGG pathway enrichment analysis of metabolites that were significantly altered in w1118 D2hgdh12–6 mutants males as compared with w1118 controls. [file media-2.pdf]

# Control vs *D2hgdh*<sup>12-6</sup>

## KEGG - Enrichment Overview (Top 25)

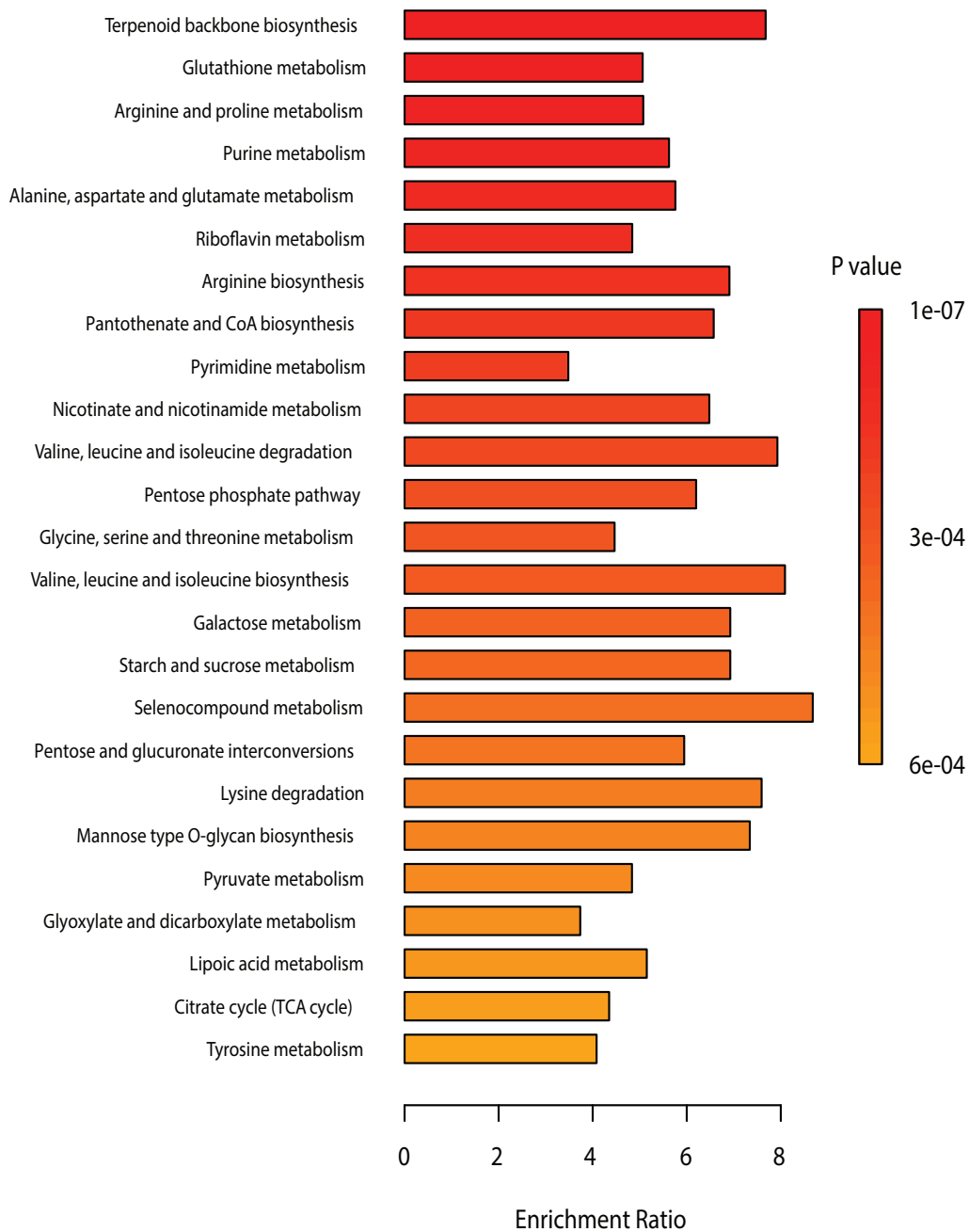

**Figure S3**

Supplement: Supplement 3 — Figure S1. Enrichment analysis of D2hgdh5–5 mutants in comparison to controls. MetaboAnalyst was used to perform KEGG pathway enrichment analysis of metabolites that were significantly altered in w1118 D2hgdh5–5 mutants males as compared with w1118 controls. [file media-3.pdf]
